# Supplementary material for: Comparative Efficacy and Safety of Tezepelumab and Other Biologics in Patients with Inadequately Controlled Asthma According to Thresholds of Type 2 Inflammatory Biomarkers: A Systematic Review and Network Meta-Analysis
Source: Cells. 2022 Feb 26;11(5):819. doi: 10.3390/cells11050819 (PMC8909778; doi:10.3390/cells11050819)
Supplement: Supplementary file 1 [file cells-11-00819-s001.zip › cells-1587359-supplementary.pdf]

Supplementary Materials

Table S1. Study lists and key inclusion criteria for the systematic review and network meta-analysis.

Study name [ref]	Type of study	Phase	Study duration	PE	Key inclusion criteria
MENSA 2014 [43]	RCT DB, PC	Ⅲ	32 weeks	・AER	・Aged 12 to 82 years<br>・At least two asthma exacerbations in the previous year<br>・Received at least 880 μg of fluticasone propionate or the equivalent by inhalation/day<br>・Eosinophil count of at least 150 cells/μL in the peripheral blood at screening or at least 300 cells/μL at some point during the previous year.
CALIMA 2016 [46]	RCT DB, PC	Ⅲ	56 weeks	・AER	・Aged 12 to 75 years<br>・Two or more asthma exacerbations in the 12 months before enrollment<br>・Required treatment with medium-dosage to high-dosage ICS (>250 μg [medium] or ≥500 μg [high] fluticasone dry powder formulation or equivalent total daily dosage) plus LABA.
SIROCCO 2016 [45]	RCT DB, PC	Ⅲ	48 weeks	・AER	・Aged 12 to 75 years<br>・At least two documented asthma exacerbations within 1 year before enrollment<br>・Needed treatment with medium-dosage or high- dosage ICS plus LABA for at least 1 year before enrollment

MUSCA 2017 [44]	RCT DB, PC	Ⅲb	24 weeks	・SGRQ score	・12 years of age or older<br>・Experienced at least two exacerbations in the previous 12 months<br>・Received treatment with regular high-dose ICS in the 12 months before screening plus additional controller medications for at least 3 months before screening.<br>・Blood eosinophil count of at least 300 cells/μL 12 months before screening or a blood eosinophil count of at least 150 cells/μL at the time of screening
LIVERTY ASTHMA QUEST 2018 [49]	RCT DB, PC	Ⅲ	52 weeks	・AER<br>・Change in pre-BD $FEV_{1.0}$	・12 years of age or older<br>・ A worsening of asthma in the previous year<br>・Current treatment with a medium-to-high-dose ICS (fluticasone propionate at a total daily dose of ≥500 μg or equipotent equivalent) plus up to two additional controllers.
SOLANA 2020 [47]	RCT DB, PC	Ⅲb	12 weeks	・Change in pre-BD $FEV_{1.0}$	・Aged 18 to 75 years<br>・At least two documented asthma exacerbations within 12 months before enrollment.<br>・Required treatment with ICS/LABA for ≥30 days before enrollment<br>・Peripheral blood eosinophil count ≥300 cells/μL
ANDHI 2021 [48]	RCT DB, PC	Ⅲb	24 weeks	・AER	・Aged 18 to 75 years<br>・At least two asthma exacerbations within 12 months before enrollment.

					・Received treatment with high-dose ICS plus another asthma controller for 3 months before enrollment.<br>・Peripheral blood eosinophil count ≥300 cells/μL
NAVIGATOR 2021 [15]	RCT DB, PC	Ⅲ	52 weeks	・AER	・Aged 12 to 80 years<br>・At least two asthma exacerbations in the 12 months<br>・Received medium- or high-dose ICS (daily dose of ≥500 μg of fluticasone propionate or equivalent)

PE, primary endpoint; RCT, randomized controlled trial; DB, double blind; PC, placebo controlled; AER, annualized exacerbation rate; ICS, inhaled corticosteroid; LABA, long acting beta-2 antagonist; SGRQ, St. George's Respiratory Questionnaire; pre-BD $FEV_{1.0}$, pre-bronchodilator forced expiratory volume in one second.

Table S2. Characteristics of the included studies.

Study name y [ref]	Treatment arms	No.	Age-y*	Female No. (%)	BMI (kg/m²)	Pre-BD $FEV_{1.0}$ (L)	PBEC† (cells/mm³)	FeNO (ppb)	ACQ score‡	AQLQ score§	AE¶
MENSA 2014 [43]	Mepo 100 mg s.c., e4w	194	51 (12–81)	116 (60%)	27.6 (6.2)	1.73 (0.66)	290 (1050)	NR	2.26 (1.27)	NR	3.8 (2.7)
	Placebo e4w	191	49 (12–76)	107 (56%)	28.0 (5.6)	1.86 (0.63)	320 (938)	NR	2.28 (1.19)	NR	3.6 (2.8)
		385/total									
CALIMA 2016 [46]	Benr 30 mg s.c., e8w (e4w, at f3d)	441	49.0 (14.3)	273 (62%)	28.8 (6.5)	1.759 (0.641)	400 (0–2600)	NR	2.75 (0.93)	3.85 (1.02)	2.7 (1.4)
	Placebo s.c., e8w (e4w, at f3d)	440	48.8 (15.1)	264 (60%)	28.9 (6.5)	1.771 (0.645)	371 (0–4494)	NR	2.69 (0.92)	3.96 (1.03)	2.7 (1.6)
		881/total									
SIROCCO 2016 [45]	Benr 30 mg s.c., e8w (e4w, at f3d)	398	47.6 (14.5)	252 (63%)	28.2 (6.2)	1.680 (0.582)	360 (0–3100)	NR	2.80 (0.88)	3.94 (1.00)	2.8 (1.5)
	placebo s.c., e8w	407	48.7 (14.9)	269 (66%)	28.9 (7.1)	1.660 (0.584)	370 (0–2690)	NR	2.87 (0.94)	3.90 (1.02)	3.0 (1.8)

	(e4w, at f3d)										
		805/total									
MUSCA 2017 [44]	Mepo 100 mg s.c., e4w	274	49.8 (14.0)	149 (54%)	28.5 (6.6)	1.8 (0.6)	300	NR	2.2 (1.1)	NR	2.9 (1.9)
	Placebo s.c., e4w	277	52.1 (12.9)	176 (64%)	27.9 (6.2)	1.7 (0.6)	350	NR	2.2 (1.2)	NR	2.7 (1.5)
		551/total									
LIVERTY ASTHMA QUEST 2018 [49]	Dupi 300 mg s.c., e2w	633	47.7 (15.6)	394 (62.2%)	29.07 (6.68)	1.78 (0.60)	351 (369)	34.01 (29.74)	2.77 (0.76)	4.28 (1.05)	2.02 (1.86)
	placebo s.c., e2w	321	48.2 (14.7)	218 (67.9%)	29.21 (6.95)	1.75 (0.57)	391 (419)	38.39 (38.00)	2.77 (0.77)	4.30 (1.03)	2.31 (2.07)
		954/total									
SOLANA 2020 [47]	Benr 30 mg s.c., e8w (e4w, at f3d)	118	51.9 (13.62)	74 (63%)	26.70 (4.56)	1.740 (0.636)	434.3 (233.44)	NR	2.65 (0.88)	NR	2.5 (1.27)
	Placebo s.c., e8w (e4w, at f3d)	115	50.9 (12.34)	83 (72%)	28.65 (6.43)	1.655 (0.556)	441.9 (258.29)	NR	2.61 (0.89)	NR	2.4 (0.83)
		232/total									
ANDHI	Benr 30 mg	427	52.5 (12.7)	263 (62%)	29.85 (7.37)	1.630 (0.609)	390 (40–7970)	NR	3.04 (0.874)	NR	3.2

2021<br>[48]	s.c., e8w<br>(e4w, at f3d)										
	Placebo<br>s.c., e8w<br>(e4w, at f3d)	229	53.3 (12.5)	136 (59%)	30.10 (7.89)	1.720 (0.629)	390 (20–5600)	NR	3.07 (0.965)	NR	3.1
		656/total									
NAVIGATOR<br>2021<br>[15]	Teze 210 mg<br>s.c., e4w	528	49.9 (16.3)	335 (63.4%)	28.7 (7.1)	1.8 (0.7)	327 (293)	41.4 (36.3)	2.8 (0.8)	3.9 (1.0)	0: 0<br>1: 0<br>2: 310 (58.7)<br>>2: 218 (41.3)
	Placebo<br>e4w	531	49.0 (15.9)	337 (63.5%)	28.3 (6.9)	1.9 (0.7)	353 (488)	46.3 (44.7)	2.8 (0.8)	3.9 (1.0)	0: 0<br>1: 1 (0.2)<br>2: 324 (61.0)<br>>2: 206 (38.8)
		1059/total									

Data are expressed as mean (SD), median (range), or n (%).

Note: *, In MENSA, Age-year was expressed as mean (range); †, In MENSA and MUSCA, PBEC was expressed as geometric means (SD); ‡, The MENSA, MUSCA, and LIVERTY ASTHMA QUEST provided mean (SD) of the ACQ-5 scores, and the other five studies (CALIMA, SIROCCO, SOLANA, ANDHI, and NAVIGATOR) provided mean (SD) of the ACQ-6 scores; §, CALIMA, SIROCCO, and NAVIGATOR provided the mean (SD) of the AQLQ(S)+12 score, and the LIVERTY ASTHMA QUEST provides the mean (SD) of the AQLQ global score; ¶, In NAVIGATOR, the no. of patients with 0, 1, 2, or >2 exacerbations in the past 12 months were described; y, year; ref, reference number; N, number of patients included in the treatment arm; No., number of patients; BMI, body mass index; pre-BD $FEV_{1.0}$, pre-bronchodilator forced expiratory volume in one second; PBEC, peripheral blood eosinophil count; FeNO, fractional exhaled nitric oxide; ACQ, asthma control questionnaire; AQLQ, asthma quality of life questionnaire; AQLQ(S)+12, standardized asthma quality of life questionnaire for 12 years and older; AE, number of exacerbations in the past 12 months; Mepo, mepolizumab; s.c., subcutaneous injection; e4w, every 4 weeks; NR, not reported; Benr, benralizumab; e8w, every 8 weeks; f3d, first three doses; Dupi, dupilumab; e2w, every 2 weeks; Teze, tezepelumab.

Table S3. Summary of the included studies on the basis of population for the analysis of each predefined endpoint.

	Population for analysis		Study name [ref]							
Endpoints	OP or SG	Thresholds	MEN [43]	CAL [46]	SIR [45]	MUS [44]	LIV [49]	SOL [47]	AND [48]	NAV [15]
AER	OP		○	○	○	○	○	×	○	○
	SG (by PBEC)	≥300 cells/mm³	○	○	○	×	○	×	×	○
		<300 cells/mm³	○	○	○	×	○	×	×	○
		≥150 cells/mm³	○	○	○	×	○	×	○	○
		<150 cells/mm³	○	○	○	×	○	×	×	○
	SG (by FeNO)	≥50 ppb	×	×	×	×	○	×	×	○
		<50 ppb	×	×	×	×	○	×	×	○
		≥25 ppb	×	×	×	×	○	×	×	○
		<25 ppb	×	×	×	×	○	×	×	○
Change in Pre-BD $FEV_{1.0}$	OP		○	○	○	○	○	○	○	○
	SG (by PBEC)	≥300 cells/mm³	×	○	○	×	○	○	○	○
		<300 cells/mm³	×	○	○	×	○	×	×	○
		≥150 cells/mm³	×	○	○	×	○	○	○	○
		<150 cells/mm³	×	○	○	×	○	×	×	○
Change in AQLQ score	OP		×	○	○	×	○	×	×	○
	SG (by PBEC)	≥300 cells/mm³	×	○	○	×	○	×	×	○
		≥150 cells/mm³	×	○	○	×	×	×	×	○

Change in ACQ score	OP		○	○	○	○	○	○	○	○
	SG (by PBEC)	≥300 cells/mm³	×	○	○	×	×	○	○	○
		<300 cells/mm³	×	○	○	×	×	×	×	○
		≥150 cells/mm³	×	○	○	×	×	○	○	○
AAEs	OP		○	○	○	○	○	○	○	○

A summary of the included studies by population for the analysis of each predefined endpoint is expressed. The symbols "○"and "×" indicate inclusion (outcome data required for the relevant analysis have been reported) and not inclusion (outcome data required for the relevant analysis have not been reported) in the analysis of the corresponding population for the relevant endpoint, respectively; ref, reference; OP, overall population, SG, subgroup; MEN, MENSA; CAL, CALIMA; SIR, SIROCCO; MUS, MUSCA, LIV, LIVERTY ASTHMA QUEST, SOL, SOLANA; AND, ANDHI; NAV, NAVIGATOR; AER, annualized exacerbation rate; PBEC, peripheral blood eosinophil count; FeNO, fractional nitric oxide concentration in exhaled breath; pre-BD $FEV_{1.0}$, pre-bronchodilator forced expiratory volume in one second; AQLQ, Asthma Quality of Life Questionnaire; ACQ, Asthma Control Questionnaire, AAEs, any adverse events.

Table S4. Comparative efficacy of each pair of the five treatment arms, including tezepelumab, dupilumab, benralizumab, mepolizumab, and placebo with respect to AER in overall population and in subgroups by PBEC thresholds.

Treatment comparison		Overall	PBEC thresholds			
			≥300 cells/mm³	<300 cells/mm³	≥150 cells/mm³	<150 cells/mm³
Teze vs.	Plac	0.440 (0.368–0.526)*	0.300 (0.222–0.404)*	0.590 (0.462–0.753)*	0.390 (0.315–0.482)*	0.610 (0.421–0.881)*
	Mepo	0.991 (0.751–1.310)	0.960 (0.569–1.624)	0.939 (0.557–1.580)	1.004 (0.660–1.528)	1.015 (0.487–2.115)
	Benr	0.734 (0.588–0.916)*	0.508 (0.356–0.725)*	0.828 (0.586–1.169)	0.659 (0.512–0.850)*	0.847 (0.497–1.440)
	Dupi	0.815 (0.609–1.092)	0.909 (0.581–1.429)	0.714 (0.479–1.067)	0.987 (0.699–1.398)	0.531 (0.302–0.939)*
Dupi vs.	Plac	0.540 (0.429–0.679)*	0.330 (0.236–0.461)*	0.826 (0.602–1.133)	0.395 (0.301–0.519)*	1.148 (0.747–1.764)
	Mepo	1.216 (0.889–1.662)	1.056 (0.611–1.823)	1.316 (0.751–2.300)	1.017 (0.646–1.599)	1.913 (0.887–4.114)
	Benr	0.901 (0.692–1.172)	0.558 (0.379–0.824)*	1.160 (0.778–1.731)	0.668 (0.492–0.907)*	1.595 (0.898–2.847)
Benr vs.	Plac	0.599 (0.526–0.683)*	0.591 (0.485–0.718)*	0.713 (0.557–0.910)*	0.592 (0.514–0.680)*	0.720 (0.490–1.057)
	Mepo	1.350 (1.052–1.732)*	1.891 (1.179–3.027)*	1.134 (0.673–1.908)	1.522 (1.033–2.238)*	1.198 (0.571–2.510)
Mepo vs.	Plac	0.444 (0.359–0.549)*	0.312 (0.203–0.481)*	0.628 (0.397–0.997)*	0.389 (0.271–0.559)*	0.601 (0.320–1.135)

Note: *, statistically significant (95% CrI does not include 1)
The comparative efficacy of each pair of the five treatment arms, including tezepelumab, dupilumab, benralizumab, mepolizumab, and placebo with respect to AER in the overall population, and in subgroups with PBEC of ≥300, <300, ≥150, and <150 cells/mm³. The included studies in each of the patient populations analyzed (overall populations and subgroups with PBEC of ≥300, <300, ≥150, and <150 cells/mm³) are detailed in Table S3. Data are expressed as rate ratios (RRs) and 95% credible intervals (CrIs). The effect size is expressed as Treatment A vs. Treatment B. If the 95% CrI value does not include 1, the difference in effect size between the drugs is considered to be significant. If the 95% CrI is localized in the range less than 1, Treatment A is more effective than Treatment B in terms of AER, whereas if the 95% CrI is located in the range greater than 1, Treatment A is less effective than Treatment B in terms of AER. For example, tezepelumab was found to be significantly more effective than benralizumab in the overall group and in the groups with PBEC ≥300 and ≥150, and more effective than dupilumab in the group with PBEC <150. The results of the comparison between tezepelumab and dupilumab agents for AER are presented visually in Figure 4 of the main manuscript file. AER, annualized exacerbation rate; PBEC, peripheral blood eosinophil count; Teze, tezepelumab; Dupi, dupilumab; Benr, benralizumab; Mepo, mepolizumab; Plac, placebo.

Table S5. SUCRA (rank) for AER in the overall population and in subgroups according to PBEC thresholds.

Interventions	Overall	PBEC thresholds			
		≥300 cells/mm^3	<300 cells/mm^3	≥150 cells/mm^3	<150 cells/mm^3
Plac	0.0 (5)	0.0 (5)	3.7 (5)	0.0 (5)	21.2 (4)
Mepo	83.8 (2)	75.4 (2)	72.4 (2)	75.5 (2)	77.4 (2)
Benr	30.8 (4)	25.2 (4)	55.6 (3)	25.6 (4)	62.1 (3)
Dupi	49.4 (3)	69.0 (3)	33.3 (4)	73.4 (3)	9.6 (5)
Teze	86.0 (1)	80.5 (1)	85.0 (1)	75.6 (1)	79.9 (1)

Data presented are the surface under the cumulative ranking curve (SUCRA) values for efficacy in terms of AER for the five treatment arms (tezepelumab, dupilumab, benralizumab, mepolizumab, and placebo) in patients with inadequately controlled asthma. The included studies in each of the patient populations analyzed (overall populations and subgroups with PBEC of ≥300, <300, ≥150, and <150 cells/mm^3) are detailed in Table S3. Data are presented as SUCRA values (rank), and higher SUCRA values indicate better outcomes. It should be noted that SUCRA values alone cannot verify the significance of the difference in efficacy of the drugs, and it was therefore not possible to make a final conclusion about these differences. AER, annualized exacerbation rate; PBEC, peripheral blood eosinophil count; Teze, tezepelumab; Dupi, dupilumab; Benr, benralizumab; Mepo, mepolizumab; Plac, placebo.

Table S6. Comparative efficacy for each pair of the three treatment arms, including tezepelumab, dupilumab, and placebo, with respect to AER in subgroups according to FeNO thresholds.

Treatment comparison		FeNO thresholds			
		≥50 ppb	<50 ppb	≥25 ppb	<25 ppb
Teze vs.	Plac	0.270 (0.190–0.382)*	0.544 (0.434–0.681)*	0.320 (0.246–0.415)*	0.680 (0.505–0.914)*
	Dupi	0.870 (0.485–1.570)	0.843 (0.596–1.197)	0.858 (0.567–1.306)	0.860 (0.554–1.341)
Dupi vs.	Plac	0.310 (0.193–0.497)*	0.645 (0.494–0.841)*	0.372 (0.269–0.516)*	0.790 (0.568–1.097)

Note: *statistically significant (95% CrI does not include 1).

The included studies in each of the patient populations analyzed (subgroups with FeNO of ≥50, <50, ≥25, and <25 ppb) are detailed in Table S3. Data are expressed as rate ratios (RRs) and 95% credible intervals (CrIs). The effect size is expressed as Treatment A vs. Treatment B. If the 95% CrI value does not include 1, the difference in effect size between the drugs is considered to be significant. If the 95% CrI is localized in the range less than 1, Treatment A is more effective than Treatment B in terms of AER, whereas if the 95% CrI is located in the range greater than 1, Treatment A is less effective than Treatment B in terms of AER. The results of the comparison between tezepelumab and dupilumab agents for AER are presented visually in Figure 4 of the main manuscript file. AER, annualized exacerbation rate; FeNO, fractional exhaled nitric oxide; Teze, tezepelumab; Dupi, dupilumab; Plac, placebo.

Table S7. SUCRA (rank) for AER in subgroups according to FeNO thresholds.

Interventions	PBEC thresholds			
	≥50 ppb	<50 ppb	≥25 ppb	<25 ppb
Plac	0.0 (3)	0.0 (3)	0.0 (3)	4.3 (3)
Dupi	66.1 (2)	58.5 (2)	61.9 (2)	58.7 (2)
Teze	83.9 (1)	91.6 (1)	88.2 (1)	87.1 (1)

Data presented are the surface under the cumulative ranking curve (SUCRA) values for efficacy in terms of AER for the five treatment arms (tezepelumab, dupilumab, benralizumab, mepolizumab, and placebo) in patients with inadequately controlled asthma. The included studies in each of the patient populations analyzed (overall populations and subgroups with FeNO of ≥50, <50, ≥25, and <25) are detailed in Table S3. The data are listed as SUCRA values (rank), and higher SUCRA values indicate better outcomes. It should be noted that SUCRA values alone cannot verify the significance of the difference in efficacy of the drugs, and it was therefore not possible to make a final conclusion about these differences. AER, annualized exacerbation rate; FeNO, fractional exhaled nitric oxide; Teze, tezepelumab; Dupi, dupilumab; Benr, benralizumab; Mepo, mepolizumab; Plac, placebo.

Table S8. Comparative efficacy for each pair of five treatment arms, including tezepelumab, dupilumab, benralizumab, mepolizumab, and placebo, with respect to the change in pre-BD $FEV_{1.0}$ in the overall population and in subgroups according to PBEC thresholds.

Treatment comparison		Overall	PBEC thresholds			
			≥300 cells/mm³	<300 cells/mm³	≥150 cells/mm³	<150 cells/mm³
Teze vs.	Plac	0.130 (0.080–0.180)*	0.230 (0.149–0.310)*	0.070 (0.004–0.135)*	0.170 (0.109–0.230)*	0.030 (−0.071–0.129)
	Mepo	0.019 (−0.056–0.094)	NE	NE	NE	NE
	Benr	0.023 (−0.038–0.084)	0.101 (0.010–0.191)*	0.022 (−0.079–0.122)	0.053 (−0.018–0.124)	−0.014 (−0.163–0.134)
	Dupi	0.000 (−0.071–0.071)	−0.010 (−0.124–0.103)	0.022 (−0.073–0.117)	0.023 (−0.064–0.110)	−0.060 (−0.199–0.077)
Dupi vs.	Plac	0.130 (0.080–0.180)*	0.240 (0.160–0.321)*	0.047 (−0.021–0.117)	0.146 (0.084–0.209)*	0.090 (−0.005–0.186)
	Mepo	0.019 (−0.056–0.094)	NE	NE	NE	NE
	Benr	0.023 (−0.038–0.084)	0.111 (0.021–0.202)*	−0.001 (−0.103–0.101)	0.029 (−0.043–0.102)	0.046 (−0.098–0.191)
Benr vs.	Plac	0.107 (0.071–0.142)*	0.129 (0.087–0.171)*	0.048 (−0.027–0.124)	0.117 (0.080–0.154)*	0.044 (−0.065–0.153)
	Mepo	−0.004 (−0.070–0.062)	NE	NE	NE	NE
Mepo vs.	Plac	0.111 (0.055–0.167)*	NE	NE	NE	NE

Note: *statistically significant (95% CrI does not include 0).

The included studies in each of the patient populations analyzed (overall populations and subgroups with PBEC of ≥300, <300, ≥150, and <150 cells/mm³) are detailed in Table S3. Data are expressed as mean differences (MDs) and 95% credible intervals (CrIs). The effect size is expressed as Treatment A vs. Treatment B. If the 95% CrI value does not include 0, the difference in effect size between the drugs is considered to be significant. If the 95% CrI is localized in the range greater than 0, Treatment A is more effective than Treatment B in terms of pre-BD $FEV_{1.0}$, whereas if the 95% CrI is located in the range less than 0, Treatment A is less effective than Treatment B in terms of pre-BD $FEV_{1.0}$. The results of the comparison between tezepelumab and dupilumab agents for changes in pre-BD $FEV_{1.0}$ are also presented visually in Figure 5 of the main manuscript file; pre-BD $FEV_{1.0}$, pre-bronchodilator forced expiratory volume in one second; PBEC, peripheral blood eosinophil counts; Teze, tezepelubmab; Dupi, dupilumab; Benr, benralizumab; Mepo, mepolizumab; Plac, placebo; NE, not evaluable.

Table S9. SUCRA (rank) for the change in pre-BD $FEV_{1.0}$ in the overall population and in subgroups according to PBEC thresholds.

Interventions	Overall	PBEC thresholds			
		≥300 cells/mm³	<300 cells/mm³	≥150 cells/mm³	<150 cells/mm³
Plac	0.0 (5)	0,0 (4)	7.1 (4)	0.0 (4)	17.5 (4)
Mepo	54.2 (3)	NE	NE	NE	NE
Benr	47.8 (4)	34.1 (3)	57.9 (2)	42.9 (3)	54.1 (2)
Dupi	74.0 (2)	85.5 (1)	57.5 (3)	69.5 (2)	83.6 (1)
Teze	74.1 (1)	80.5 (2)	77.5 (1)	87.7 (1)	44.8 (3)

The data presented are the surface under the cumulative ranking curve (SUCRA) values for efficacy in terms of the change in pre-BD $FEV_{1.0}$ for the five treatment arms (tezepelumab, dupilumab, benralizumab, mepolizumab, and placebo) in patients with inadequately controlled asthma. The included studies in each of the patient populations analyzed (overall populations and subgroups with PBEC of ≥300, <300, ≥150, and <150 cells/mm³) are detailed in Table S3. The data are listed as SUCRA values (rank), and higher SUCRA values indicate better outcomes. It should be noted that SUCRA values alone cannot verify the significance of the difference in efficacy of the drugs, and it was therefore not possible to make a final conclusion about these differences. ; pre-BD $FEV_{1.0}$, pre-bronchodilator forced expiratory volume in one second; PBEC, peripheral blood eosinophil counts; Teze, tezepelumab; Dupi, dupilumab; Benr, benralizumab; Mepo, mepolizumab; Plac, placebo; NE, not evaluable.

Table S10. Comparative efficacy for each pair of the five treatment arms, including tezepelumab, dupilumab, benralizumab, mepolizumab, and placebo, with respect to the change in AQLQ score in the overall population and in subgroups according to PBEC thresholds.

Treatment comparison		Overall	PBEC thresholds	
			≥300 cells/mm³	≥150 cells/mm³
Teze vs.	Plac	0.300 (0.179–0.419)*	0.460 (0.273–0.644)*	0.380 (0.234–0.525)*
	Mepo	NE	NE	NE
	Benr	0.110 (−0.047–0.266)	0.200 (−0.024–0.423)	0.199 (0.018–0.382)*
	Dupi	0.120 (−0.062–0.300)	0.219 (−0.059–0.495)	NE
Dupi vs.	Plac	0.180 (0.045–0.316)*	0.240 (0.036–0.447)*	NE
	Mepo	NE	NE	NE
	Benr	−0.010 (−0.177–0.159)	−0.020 (−0.259–0.222)	NE
Benr vs.	Plac	0.190 (0.090–0.290)*	0.260 (0.135–0.385)*	0.180 (0.070–0.290)*
	Mepo	NE	NE	NE
Mepo vs.	Plac	NE	NE	NE

Note: *statistically significant (95% CrI does not include 0)

Comparative efficacy of each pair of the four treatment arms, including tezepelumab, dupilumab, benralizumab, and placebo, with respect to changes in the AQLQ score in the overall population and in subgroups with PBEC of ≥300 and ≥150 cells/mm³. The mepolizumab study did not report outcome data for the change in AQLQ scores; accordingly, it was not included in this analysis. The included studies in each of the patient populations analyzed (overall populations and subgroups with PBEC of ≥300 and ≥150 cells/mm³) are detailed in Table S3. Data are expressed as standardized mean differences (SMDs) and 95% credible intervals (CrIs). The effect size is expressed as Treatment A vs. Treatment B. If the 95% CrI value does not include 0, the difference in effect size between the drugs is considered to be significant. If the 95% CrI is localized in the range greater than 0, Treatment A is more effective than Treatment B in terms of change in AQLQ score, whereas if the 95% CrI is located in the range less than 0, Treatment A is less effective than Treatment B in terms of change in AQLQ score. The results of the comparison between tezepelumab

and dupilumab agents are also presented visually in Figure 6 of the main manuscript file. AQLQ, asthma quality of life questionnaire; PBEC, peripheral blood eosinophil counts; Teze, tezepelumab; Dupi, dupilumab; Benr, benralizumab; Mepo, mepolizumab; Plac, placebo; NE, not evaluable.

Table S11. SUCRA (rank) for changes in the AQLQ score in the overall population and in subgroups according to PBEC thresholds.

Interventions	Overall	PBEC thresholds	
		≥300 cells/mm^3	≥150 cells/mm^3
Plac	0.1 (4)	0.3 (4)	0.0 (3)
Benr	54.3 (2)	53.4 (2)	50.8 (2)
Dupi	51.6 (3)	49.6 (3)	NE
Teze	93.9 (1)	96.7 (1)	99.2 (1)

The data presented are the surface under the cumulative ranking curve (SUCRA) values for efficacy in terms of changes in the AQLQ score for the four treatment arms (tezepelumab, dupilumab, benralizumab, and placebo) in patients with inadequately controlled asthma. The mepolizumab study did not report outcome data for the change in AQLQ score; accordingly, it was not included in this analysis. The included studies in each of the patient populations analyzed (overall populations and subgroups with PBEC of ≥300 and ≥150 cells/mm^3) are detailed in Table S3. The data are listed as SUCRA values (rank), and higher SUCRA values indicate better outcomes. It should be noted that SUCRA values alone cannot verify the significance of the difference in efficacy of the drugs, and it was therefore not possible to make a final conclusion about these differences. AQLQ, asthma quality of life questionnaire; PBEC, peripheral blood eosinophil counts; Teze, tezepelumab; Dupi, dupilumab; Benr, benralizumab; Plac, placebo; NE, not evaluable.

Table S12. Comparative efficacy for each pair of five treatment arms, including tezepelumab, dupilumab, benralizumab, mepolizumab, and placebo, with respect to changes in the ACQ score in the overall population and in subgroups according to PBEC thresholds.

Treatment comparison		Overall	PBEC thresholds		
			≥300 cells/mm³	<300 cells/mm³	≥150 cells/mm³
Teze vs.	Plac	−0.310 (−0.430– −0.191)*	−0.500 (−0.692– −0.310)*	−0.210 (−0.372– −0.050)*	−0.410 (−0.556– −0.265)*
	Mepo	0.080 (−0.097–0.258)	NE	NE	NE
	Benr	−0.069 (−0.214–0.075)	−0.184 (−0.397–0.031)	−0.059 (−0.294–0.179)	−0.184 (−0.352– −0.014)*
	Dupi	−0.160 (−0.337–0.018)	NE	NE	NE
Dupi vs.	Plac	−0.151 (−0.281– −0.020)*	NE	NE	NE
	Mepo	0.240 (0.055–0.424)*	NE	NE	NE
	Benr	0.091 (−0.062–0.244)	NE	NE	NE
Benr vs.	Plac	−0.241 (−0.322– −0.160)*	−0.317 (−0.415– −0.220)*	−0.152 (−0.326–0.021)	−0.227 (−0.313– −0.141)*
	Mepo	0.149 (−0.004–0.302)	NE	NE	NE
Mepo vs.	Plac	−0.390 (−0.520– −0.259)*	NE	NE	NE

Note: *statistically significant (95% CrI does not include 0).
The comparative efficacy of each pair of the five treatment arms, including tezepelumab, dupilumab, benralizumab, mepolizumab, and placebo, with respect to changes in the ACQ score in the overall population and in subgroups with PBEC of ≥300, <300, and ≥150 cells/mm³. The included studies in each of the patient populations analyzed (overall populations and subgroups with PBEC of ≥300, <300, and ≥150 cells/mm³) are detailed in Table S3. Data are expressed as standardized mean differences (SMDs) and 95% credible intervals (CrIs). The effect size is expressed as Treatment A vs. Treatment B. If the 95% CrI value does not include 0, the difference in effect size between the drugs is considered to be significant. If the 95% CrI is localized in the range less than 0, Treatment A is more effective than Treatment B in terms of change in ACQ score, whereas if the 95% CrI is located in the range greater than 0, Treatment A is less effective than Treatment B in terms of change in ACQ score. ACQ, asthma control questionnaire; PBEC, peripheral blood eosinophil count; Teze, tezepelumab; Dupi, dupilumab; Benr, benralizumab; Mepo, mepolizumab; Plac, placebo.

Table S13. SUCRA (rank) values for the change in the ACQ score in the overall population and in subgroups according to PBEC thresholds.

Interventions	Overall	PBEC thresholds		
		≥300 cells/mm^3	<300 cells/mm^3	≥150 cells/mm^3
Plac	0.3 (5)	0.0 (3)	2.4 (3)	0.0 (3)
Mepo	94.5 (1)	NE	NE	NE
Benr	52.0 (3)	52.4 (2)	63.6 (2)	50.9 (2)
Dupi	28.9 (4)	NE	NE	NE
Teze	74.3 (2)	97.7 (1)	84.0 (1)	99.2 (1)

The data presented are the surface under the cumulative ranking curve (SUCRA) values for efficacy in terms of change in ACQ score for the five treatment arms (tezepelumab, dupilumab, benralizumab, mepolizumab, and placebo) in patients with inadequately controlled asthma. The included studies in each of the patient populations analyzed (overall populations and subgroups with PBEC of ≥300, <300, and ≥150 cells/mm^3) are detailed in Table S3. The data are listed as SUCRA values (rank), and higher SUCRA values indicate better outcomes. It should be noted that SUCRA values alone cannot verify the significance of the difference in efficacy of the drugs, and it was therefore not possible to make a final conclusion about these differences. ACQ, asthma control questionnaire; PBEC, peripheral blood eosinophil count; Teze, tezepelumab; Dupi, dupilumab; Benr, benralizumab; Mepo, mepolizumab; Plac, placebo; NE, not evaluable.

Table S14. Comparative efficacy for each pair of five treatment arms, including tezepelumab, dupilumab, benralizumab, mepolizumab, and placebo, with respect to the incidence of AAEs in the overall population.

Treatment comparison		ORs
Teze vs.	Plac	0.800 (0.591–1.081)
	Mepo	1.009 (0.660–1.549)
	Benr	0.917 (0.647–1.297)
	Dupi	0.964 (0.604–1.547)
Dupi vs.	Plac	0.829 (0.578–1.187)
	Mepo	1.047 (0.655–1.674)
	Benr	0.951 (0.637–1.416)
Benr vs.	Plac	0.873 (0.733–1.037)
	Mepo	1.101 (0.778–1.557)
Mepo vs.	Plac	0.793 (0.587–1.072)

Comparative efficacy for each pair of five treatment arms, including tezepelumab, dupilumab, benralizumab, mepolizumab, and placebo, with respect to the incidence of AAEs in the overall population. All eight studies (MENSA, CALIMA, SIROCCO, MUSCA, LIVERTY ASTHMA QUEST, SOLANA, ANDHI, and NAVIGATOR) were included in the analysis. Data are expressed as odds ratios (ORs) and 95% credible intervals (CrIs). The effect size is expressed as Treatment A vs. Treatment B. If the 95% CrI value does not include 1, the difference in effect size between the drugs is considered to be significant. If the 95% CrI is localized in the range less than 0, Treatment A is safer than Treatment B in terms of incidence of AAEs, whereas if the 95% CrI is located in the range greater than 1, Treatment A is less safe than Treatment B in terms of incidence of AAEs. AAEs, any adverse events; Teze, tezepelumab; Dupi, dupilumab; Benr, benralizumab; Mepo, mepolizumab; Plac, placebo

Table S15. SUCRA (rank) values for the incidence of AAEs in the overall population.

Interventions	SUCRA (rank)
Plac	8.9 (5)
Mepo	68.4 (1)
Benr	48.7 (4)
Dupi	57.7 (3)
Teze	66.5 (2)

The data presented are the surface under the cumulative ranking curve (SUCRA) values for efficacy in terms of incidence of AAEs for the five treatment arms (tezepelumab, dupilumab, benralizumab, mepolizumab, and placebo) in patients with inadequately controlled asthma. All eight studies (MENSA, CALIMA, SIROCCO, MUSCA, LIVERTY ASTHMA QUEST, SOLANA, ANDHI, and NAVIGATOR) were included in the analysis. The data are listed as SUCRA values (rank), and higher SUCRA values indicate better outcomes. It should be noted that SUCRA values alone cannot verify the significance of the difference in efficacy of the drugs, and it was therefore not possible to make a final conclusion about these differences. AAEs, any adverse events; Teze, tezepelumab; Dupi, dupilumab; Benr, benralizumab; Mepo, mepolizumab; Plac, placebo

Table S16. Sensitivity analysis.

Treatment comparison		MD
Teze vs.	Plac	0.130 (0.080–0.180)*
	Mepo	0.019 (−0.056–0.094)
	Benr	0.010 (−0.053–0.074)
	Dupi	0.000 (−0.071–0.071)
Dupi vs.	Plac	0.130 (0.080–0.180)*
	Mepo	0.019 (−0.056–0.094)
	Benr	0.010 (−0.053–0.074)
Benr vs.	Plac	0.120 (0.080–0.159)*
	Mepo	0.009 (−0.060–0.077)
Mepo vs.	Plac	0.111 (0.055–0.167)*

Note: *statistically significant (95% CrI does not include 0).
A sensitivity analysis of the change in pre-BD $FEV_{1.0}$ was performed by excluding SOLANA. Comparative efficacy of each pair of the five treatment arms, including tezepelumab, dupilumab, benralizumab, mepolizumab, and placebo, with respect to the change in pre-BD $FEV_{1.0}$ in the overall population was performed. Seven studies (MENSA, CALIMA, SIROCCO, MUSCA, LIVERTY ASTHMA QUEST, ANDHI, and NAVIGATOR) were included in the analysis. Data are expressed as mean differences (MDs) and 95% credible intervals (CrIs) The effect size is expressed as Treatment A vs. Treatment B. If the 95% CrI value does not include 0, the difference in effect size between the drugs is considered to be significant. If the 95% CrI is localized in the range greater than 0, Treatment A is more effective than Treatment B in terms of pre-BD $FEV_{1.0}$, whereas if the 95% CrI is located in the range less than 0, Treatment A is less effective than Treatment B in terms of pre-BD $FEV_{1.0}$.; pre-BD $FEV_{1.0}$, pre-bronchodilator forced expiratory volume in one second. Teze, tezepelumab; Dupi, dupilumab; Benr, benralizumab; Mepo, mepolizumab; Plac, placebo.

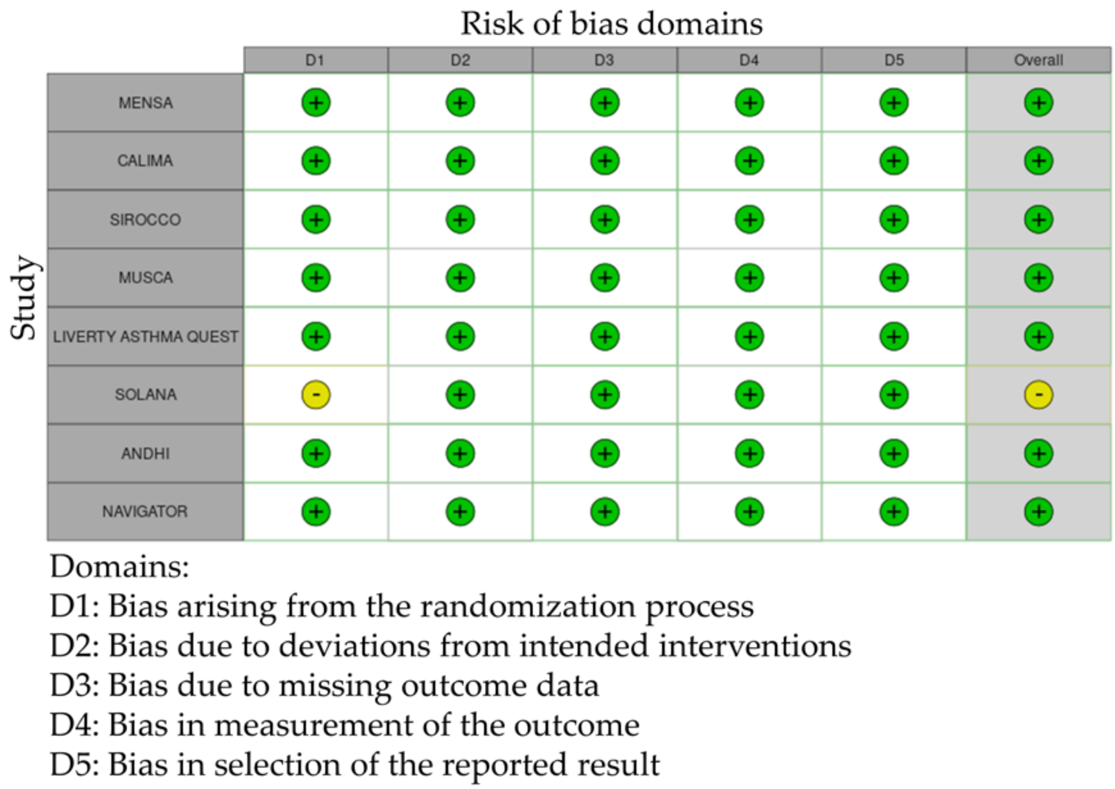

Figure S1. Risk of bias summary. The authors' assessment of each risk of bias item for each study is expressed. The symbols "+," "-," and "×" indicate a low risk of bias, some concerns, and a high risk of bias, respectively. The quality of the included studies was considered generally good, as no study was assessed as having a high risk of bias. One study [47] included in the current systematic review and NMA was judged as having some concerns in the domain of bias arising from the randomization process owing to inadequate descriptions of the details of randomization.

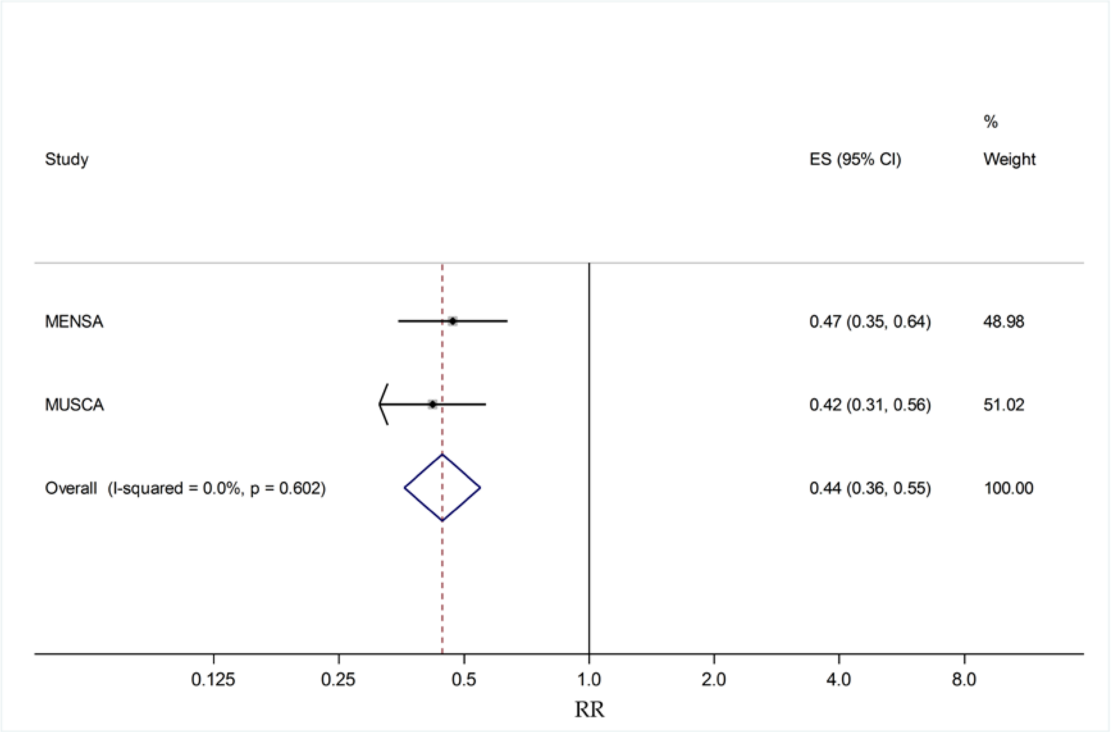

Figure S2. Forest plot for two trials comparing mepolizumab and placebo. A meta-analysis of two trials MENSA [43] and MUSCA [44]) comparing AER among groups was performed based on a random effect model, with an assessment of heterogeneity (I^2), expressed as a percentage. Overall effect size (ES) for AER was expressed as RR (mepolizumab versus placebo) and 95% CI. The data were obtained from previous studies [43, 44]. RR, rate ratio; CI, confidence interval; AER, annualized exacerbation rate.

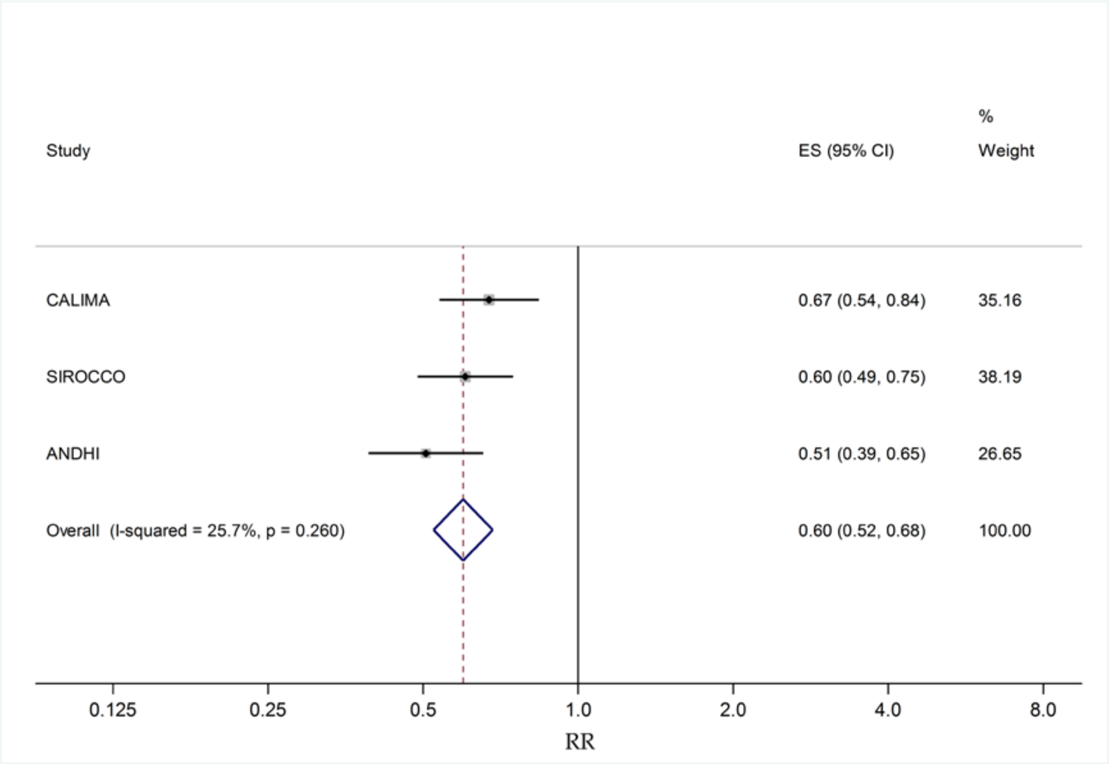

Figure S3. Forest plot for three trials comparing benralizumab and placebo. A meta-analysis of three trials CALIMA [46], SIROCCO [45], and ANDHI [48]) comparing AER among groups was performed based on a random effect model, with an assessment of heterogeneity (I^2), expressed as a percentage. Overall effect size (ES) for AER is expressed as RR (benralizumab versus placebo) and 95% CI. The data were obtained from previous studies [45, 46, 48]. In CALIMA and SIROCCO, the AER results were reported for each eosinophil count threshold but not for the overall population. Therefore, the reported data were combined to estimate the results for the overall population; RR, rate ratio; CI, confidence interval; AER, annualized exacerbation rate.
